# Using the Preparation Phase of the Multiphase Optimization Strategy to Design an Antiextremism Program in Bahrain: Formative and Pilot Research

**DOI:** 10.2196/58322

**Published:** 2024-07-17

**Authors:** Kelly Rulison, GracieLee Weaver, Jeffrey Milroy, Emily Beamon, Samantha Kelly, Ali Ameeni, Amina Juma, Fadhel Abualgasim, Jaafar Husain, David Wyrick

**Affiliations:** 1 Prevention Strategies Greensboro, NC United States; 2 Department of Public Health Education University of North Carolina Greensboro Greensboro, NC United States; 3 Center for Athlete Well-being University of North Carolina Greensboro Greensboro, NC United States; 4 D.A.R.E. Bahrain Southern Province Bahrain

**Keywords:** Antiextremism, peaceful coexistence, intervention, evaluation, international, multiphase optimization strategy, preparation phase, extremism, extremist, peace, peaceful, resistance, violent, violence, radical, radicalism, Bahrain, education, educational, school, schools, student, students, drug, drugs, abuse, substance

## Abstract

**Background:**

Extremism continues to raise concerns about conflict and violent attacks that can lead to deaths, injuries, trauma, and stress. Adolescents are especially vulnerable to radicalization by extremists. Given its location in a region that often experiences extremism, Bahrain developed 4 peaceful coexistence lessons and 4 antiextremism lessons to be implemented as part of their Drug Abuse Resistance Education (D.A.R.E.) program.

**Objective:**

The aim of this study is to report the results of the preparation phase of the multiphase optimization strategy (MOST) to develop a peaceful coexistence program and an antiextremism program implemented by D.A.R.E. officers in Bahrain.

**Methods:**

We developed conceptual models for the peaceful coexistence and antiextremism programs, indicating which mediators each lesson should target, the proximal outcomes that should be shaped by these mediators, and the distal and ultimate outcomes that the intervention should change. We recruited 20 middle schools to pilot test our research protocol, survey measures, and the existing intervention lessons. A total of 854 seventh and ninth grade students completed a pretest survey, 4 peaceful coexistence intervention lessons, and an immediate posttest survey; and a total of 495 ninth grade students completed the pretest survey, 4 antiextremism lessons, and an immediate posttest survey. A series of 3-level models, nesting students within classrooms within schools, tested mean differences from pretest to posttest.

**Results:**

Pilot test results indicated that most measures had adequate reliability and provided promising evidence that the existing lessons could change some of the targeted mediators and proximal outcomes. Specifically, students who completed the peaceful coexistence lessons reported significant changes in 5 targeted mediating variables (eg, injunctive norms about intolerance, *P*<.001) and 3 proximal outcomes [eg, social skills empathy (*P*=.008); tolerance beliefs (*P*=.041)]. Students who completed the antiextremism lessons reported significant changes in 3 targeted mediators [eg, self-efficacy to use resistance skills themselves (*P*<.001)], and 1 proximal outcome (ie, social skills empathy, *P*<.001).

**Conclusions:**

An effective antiextremism program has the potential to protect youth from radicalization and increase peaceful coexistence. We used the preparation phase of MOST to (1) develop a conceptual model, (2) identify the 4 lessons in each program as the components we will evaluate in the optimization phase of MOST, (3) pilot test the existing lessons, our newly developed measures, and research protocol, and (4) determine that our optimization objective will be all effective components. We will use these results to revise the existing lessons and conduct optimization trials to evaluate the efficacy of the individual lessons.

## Introduction

From 2010 to 2020, almost 115,000 terrorist attacks occurred around the world, accounting for over 266,000 deaths [1]. Although terrorist attacks have decreased since a peak in 2014, they remain 1.75 times higher than they were in 2010 and deaths associated with terrorist attacks were almost 3 times higher in 2020 than they were in 2010 [1]. Yet terrorist attacks are just one form of violent extremism, albeit one with fatal consequences that garner a lot of attention on the problems of extremism. Along with the fatal outcome of death, extremism and terrorism increase risks to physical and psychological safety through injuries, abuses, trauma, and stress [2-4].

Extremism is defined by the International Center for Cooperation and Conflict Resolution as beliefs, attitudes, feelings, actions, or strategies of someone far removed from the ordinary [2] and by the Counter Extremism Project as holding extreme political or religious beliefs [3]. Although extremist views can highlight concerns of marginalized communities that might otherwise go unnoticed, they can also lead to violent acts that harm the health, safety, and well-being of others [2,3]. Critical policy and governmental strategies are often used to address extremism, yet countering extremism requires a multifaceted approach, intervening across multiple levels of the social ecological model to prevent and resolve conflicts before violence occurs [4]. Some strategies require intensive, early efforts to promote peace-building opportunities and improve conflict resolution skills [2,5], suggesting a strong need for community-based, individual-level interventions to promote the value of peaceful coexistence and prevent extreme or violent behaviors.

The Kingdom of Bahrain is in the Middle East and North Africa (MENA) region, which experienced over 35% of all terrorist attacks from 2010 to 2020 [1]. At the political level, Bahrain has used legislation, law enforcement, and international and regional cooperation to counter and prevent extremism (eg, addressing financing of terrorism and violent extremism). Yet the government also wanted to focus on early prevention of violent extremism, creating an intervention for adolescents, who are particularly vulnerable to radicalization and recruitment into extremist groups due to their developmental exploration of identity and belonging [6,7]. In response, the Bahraini government developed a school-based intervention to prevent middle and high school students from being recruited into extremist groups and to prevent intolerance within its communities. By implementing the intervention within schools, the Bahraini government could reach a broad audience of youth and ensure all students were exposed to the same content [8].

To develop school-based interventions focused on peaceful coexistence and antiextremism, Bahrain worked collaboratively with the Drug Abuse Resistance Education (D.A.R.E.) organization. D.A.R.E. implements comprehensive K-12 prevention programs in dozens of countries around the world [9]. As implied by its name, the goal of the core D.A.R.E. curriculum is to prevent drug use. However, D.A.R.E. also works closely with its international partners to develop intervention lessons that can address the specific needs of the host country, such as violence, bullying, internet safety, and in the case here, political extremism and intolerance. In Bahrain, the D.A.R.E. program is known as ‘Ma’an,’ and, as in the United States, the lessons are taught by law enforcement officers who are specially trained in prevention and education.

Typically, once researchers develop these intervention lessons, they assemble them into a multicomponent intervention “package” and evaluate this full intervention. One drawback of this approach is that researchers cannot evaluate the effect of each individual lesson, or “component.” While the full intervention package may prove effective, some components may be weak, ineffective, or even harmful. In contrast, the multiphase optimization strategy (MOST) framework [10,11] uses 3 phases to design, optimize, and evaluate interventions. As part of the first phase (i.e., preparation phase) of MOST, researchers (1) develop a conceptual model to guide the selection of components and measures for the optimization trials, (2) conduct pilot testing to ensure that the intervention is feasible, and (3) identify an optimization objective that they will pursue in the optimization phase. During the optimization phase, researchers conduct one or more optimization trials to evaluate individual intervention components using fully powered, randomized experiments. They use the results from these trials to determine which set of components meet their optimization objective identified during the preparation phase and assemble these components into an *optimized* intervention package. Finally, researchers can complete the evaluation phase to test whether this optimized intervention package is effective, using standard methods such as randomized control trials.

In the current study, we first describe the development of the conceptual model that we used to guide the optimization trials. We then report the results of a pilot study to test our procedures and protocols that we will use to conduct optimization trials of the peaceful coexistence and antiextremism D.A.R.E. lessons. As part of the pilot study, we also conducted a pretest-posttest evaluation of the existing intervention lessons on the targeted mediating variables and proximal outcomes. This pilot study is the first in a series of trials intended to optimize the peaceful coexistence and antiextremism lessons that can be implemented as part of Ma’an. The results we present here were then used to inform adaptations to the conceptual model and the protocol for the optimization phase of MOST.

## Methods

### Preparation Phase Part 1: Developing a Conceptual Model

**Figure 1:**
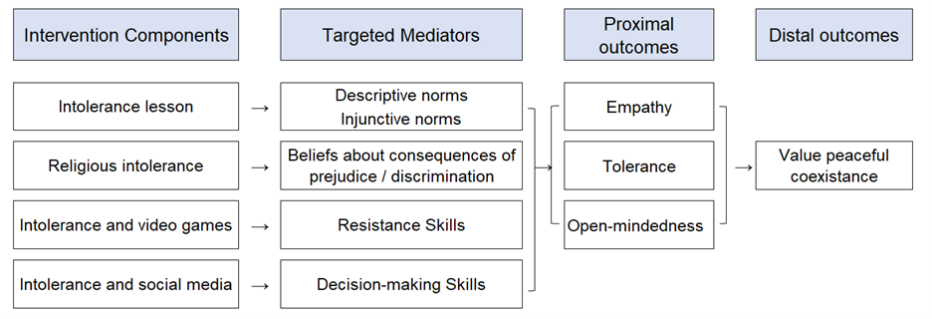
Conceptual model for anticipated effects of the peaceful coexistence lessons.

**Figure 2:**
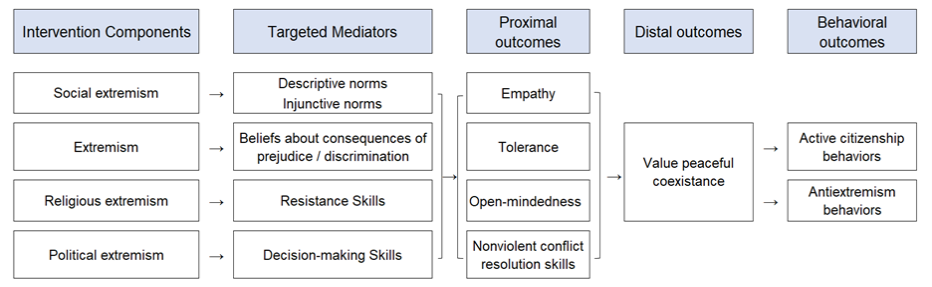
Conceptual model for anticipated effects of the antiextremism lessons.

The first goal of the preparation phase was to develop a conceptual model to identify predictors of the ultimate outcomes (here, valuing peaceful coexistence and antiextremism behaviors). Frequently, researchers create a conceptual model *before* an intervention is developed. In our case, however, the intervention content (4 lessons for each intervention) had already been created before we formed a partnership with Bahraini leaders. Therefore, to develop our conceptual model, we reviewed the current content of the lessons, conducted a rigorous review of the literature, and interviewed local stakeholders, D.A.R.E. leaders, and content experts. Based on current content, research, and stakeholder input, a preliminary conceptual model was developed and presented back to the key stakeholders and experts to obtain feedback and guidance for revisions. Following multiple rounds of revisions and input, we reached consensus on 2 conceptual models: one for the peaceful coexistence intervention lessons intended for middle school students (right 3 columns of Figure 1) and one for the antiextremism intervention lessons intended for high school students (right 3 columns of Figure 2). We then used these conceptual models to guide measurement choices and to guide future content revisions to better align with the proposed conceptual model after pilot testing.

Value of peaceful coexistence was a key distal outcome for both age groups. This focus on peaceful coexistence is particularly central to antiextremism efforts. Peaceful coexistence was recognized as an important part of international law since 1962 and stands for an agreement to do no harm without just cause, even in the face of conflict [12]. Achieving peaceful coexistence, just like antiextremism, requires multifaceted approaches, including community and individual intervention. For this reason, along with discussions and insights from the D.A.R.E. Board of Directors, leaders of Ma’an, and community officers of Bahrain, we decided to include the value of peaceful coexistence as a critical distal outcome in the conceptual model. We also extended the high school conceptual model to reflect the goal of changing *behavior* in addition to increasing students’ values of peaceful coexistence, specifically increasing active citizenship behaviors and reducing extremist or violent behaviors.

To instill a value of peaceful coexistence and antiextremism, there should be awareness of differences, acknowledging interconnectedness, embracing diversity, and practicing empathy [8,12,13]. These are reflected as proximal outcomes in both conceptual models with constructs of empathy, tolerance, and open-mindedness. Another part of peaceful coexistence is understanding and learning how to communicate in response to conflict [2,12,14], which we added as an additional proximal outcome for the high school conceptual model.

Normative beliefs is a key concept mentioned alongside peaceful coexistence education [8,12] and the one included in the core D.A.R.E drug use prevention curriculum [15,16]; thus, we included norms about intolerance as targeted mediators in both models. The core D.A.R.E curriculum has also historically aimed to change expectancies regarding consequences [15,16] and the current lessons incorporate content that aims to influence beliefs about the consequences of discrimination, included as a targeted mediator in both models. Finally, the current lessons targeted efficacy for decision-making and resistance skills, which are also targeted by the core D.A.R.E. curriculum [15,16], as they are particularly important for conflict resolution. Each of these targeted mediators has been important constructs in common behavior change theories such as the Theory of Planned Behavior [17,18] and the Social Cognitive Theory [19,20].

### Preparation Phase Part 2: Pilot Study of Existing Curriculum

#### Participants

The Bahrain Research Team recruited 20 middle schools and 20 D.A.R.E. officers (13 male; 7 female officers; 12 were high school graduates) to participate in the study. A total of 175 classrooms initially participated in the study, with a total of N=3769 seventh, eighth, and ninth grade students completing Survey 1. Officers at 4 schools (n=32 classrooms) did not administer Survey 2, 4 additional classrooms at another school were unable to complete Survey 2, and 1 classroom completed Survey 2 but had errors in the ID numbers that prevented us from matching students’ surveys to their Survey 1 data. Therefore, our final baseline sample consisted of 3090 students in 138 classrooms across 16 middle schools. Of these students, 2779 also completed Survey 2 (89.9% retention rate). A total of 1349 students in 68 classrooms received the treatment lessons and are the focus of our current analyses (see analytic plan for more details).

#### Procedures

##### Survey Development

The US Research Team developed the survey in English in collaboration with D.A.R.E.’s Director of Curriculum and Training. Then, the Bahrain Research Team reviewed the survey to adjust for unclear wording and made cultural adaptations. After the English version of the survey was finalized, the Bahrain Research Team translated the survey into Arabic and a bilingual US Research Team member reviewed the final translated survey.

##### Survey Administration

In mid-October 2021, students completed a paper or pencil version of the survey in Arabic before participating in their assigned D.A.R.E. lessons. Students took 30-45 minutes to complete Survey 1. D.A.R.E. officers then entered the data into Excel and conducted random checks for errors in data entry, sent the file to the Bahrain Research Team, who shared the file with the US Research Team. D.A.R.E. officers taught the intervention or enhancement lessons (see next section) in November and December 2021, and then approximately 1 week after the fourth lesson was taught, officers used the same process to administer Survey 2.

##### Intervention Administration

Students in seventh and eighth grade were randomly assigned to either the 4-lesson peaceful coexistence curriculum or a series of 4 enhancement lessons focusing on other topics (eg, sexual harassment and drugs). Students in ninth grade were randomly assigned to either the 4-lesson antiextremism curriculum or the same series of 4 enhancement lessons. Although the antiextremism curriculum was designed for high school students, officers implemented the program in ninth grade (middle school in Bahrain) for the pilot study because it was more difficult for D.A.R.E. to partner with new high schools and D.A.R.E. was not being implemented in enough high schools at the start of the study. The US research team created a randomization schedule, assigning individual classrooms to condition within officer. The Bahrain Research Team communicated the implementation schedule with each participating officer and the officers implemented 1 assigned lesson to each classroom each week.

### Ethical Considerations

The protocol for the current study was reviewed and approved by the IRBs at Prevention Strategies (Institutional Review Board #11690) and at the University of North Carolina at Greensboro. The Bahrain Research Team worked with schools to distribute a newsletter to parents at each middle school, which described the study and provided information about how parents could opt their child out of the study if they wanted. The first page of the student survey included a student assent form describing the study and telling students that they could skip any question they did not wish to answer. Prior to distributing the surveys to students, D.A.R.E. officers created identification numbers for students in each class and wrote these numbers on the second page of each survey. We used these ID numbers to match students’ responses over time. Students did not include any identifying information on their surveys, and they were not compensated for completing the surveys.

### Measures

#### Targeted Mediators

##### Descriptive Norms About Intolerance

Following standard measures of descriptive norms about other behaviors [21], this internally developed 7-item measure asked students “In your opinion, how many kids your age do the following?” (eg, spread hateful ideas about people who are different; insult another person’s religion; see Multimedia Appendix 1 for full list of items). Higher scores indicated students believed more children their age were acting in intolerant ways *(1 = none or almost none; 5 = all or almost all)*.

##### Injunctive Norms About Intolerance

Following standard measures of injunctive norms about other behaviors [22,23], this internally developed measure asked students “In your opinion, how do most kids your age feel about other kids doing the following?” and listed the same 7 items as above. Higher scores indicated students believed more children their age *approved* of acting in intolerant ways the mean of the 7 items *(1 = It is completely wrong; 4 = It is completely okay)*.

##### Beliefs About Consequences

This internally developed 6-item measure asked students “How likely or unlikely is it that NOT listening to different opinions will lead to each of the following?” (eg, people losing their rights; people feeling hostile toward others). Higher scores indicated students believed that ignoring different opinions was more likely to lead to negative outcomes *(1 = Very Unlikely, 4 = Very Likely).*

##### Resistance Skills Self Efficacy

We created a measure of resistance skills self-efficacy based on skills students learn in the keepin’ it REAL D.A.R.E. curriculum [24] as well as the current antiextremism and peaceful coexistence lessons: Refuse, Explain, Avoid, Leave (ie, REAL). Specifically, we asked students “If someone tried to persuade you to participate in religious, political, or socially extreme activities that you were uncomfortable with, how confident are you that you could resist (eg, say no, explain why you don’t want to participate).” Higher scores indicated greater self-efficacy to resist pressure to engage in extremist activities (*1 = Not at all confident, 4 = Completely confident)*. Students also reported their ability to encourage their friends to use these same resistance skills, using the same 4-point scale. Specifically, we asked students: “If you saw one of your friends being persuaded to participate in religious, political, or socially extreme activities they were uncomfortable with, how confident are you that YOU could...” (eg, Help your friends say no; Help your friend explain why they do not want to participate).

##### Decision-Making

We adapted a measure of resistance skills from a previous evaluation of the keepin’ it REAL substance use prevention program for elementary and middle school students [25]. In the current study, students rated the extent to which they engaged in different behaviors when they do something (eg, think carefully about their choices). We recoded items so that higher scores indicated more thoughtful or less impulsive decision-making *(1 = Strongly Disagree, 4 = Strongly Agree)*. Students also reported their ability to encourage their friends to use these same decision-making strategies, using the same 4-point scale.

#### Distal Outcomes

##### Empathy

We adapted 14 of the 15 items from the Empathy Quotient questionnaire [26] that assessed 3 factors [27]: cognitive empathy (eg, “I can quickly figure out how someone else feels), social skills (eg, “I find it hard to know what to do in a social situation”), and emotional reactivity (eg, “Seeing people cry doesn’t really upset me”). We dropped 1 item about feeling detached when watching a film and we made small modifications in wording to make the scale more developmentally appropriate. We recoded items so that each scale indicated higher empathy *(1 = Strongly Disagree, 4 = Strongly Agree).*

##### Tolerance

Our team created 10 items to assess students’ beliefs about how kids their age should act (eg, “Kids my age should have respect for other religions”). We recoded items so that higher scores indicated greater tolerance beliefs *(1 = Strongly Disagree, 4 = Strongly Agree)*.

##### Open Mindedness Toward Political and Religious Views

We asked students about their political and religious open-mindedness using 6 items adapted from Price et al [28]. Their original measures asked about politics and religion separately, but for brevity, we combined them into a single scale. Specifically, we asked students, “In your opinion, how much do YOU agree or disagree with the following statements?” (eg, “When it comes to politics and religion, I try to reserve judgment until I have a chance to hear arguments from both sides of an issue;” “I am open to considering other political or religious viewpoints”). The scale consisted of 3 positively worded (open-minded) and 3 negatively worded (ie, close-minded) items; we recoded items such that higher average scores indicated greater open-mindedness (*1 = Strongly Disagree, 4 = Strongly Agree*).

##### Open Mindedness Toward Diversity

We adapted 4 items adapted from Francis and McKenna’s [29] attitudes toward religious plurality scale (eg, All religious groups in Bahrain should have equal rights) and 2 items from their living with cultural diversity scale (ie, “People who have different religious beliefs make my school an interesting place” and “People who come from different countries make my school an interesting place”). We recoded items such that higher scores indicated greater open mindedness toward plurality or diversity (*1 = Strongly Disagree, 4 = Strongly Agree*).

##### Nonviolent Conflict Resolution

We created 6 items to capture students’ attitudes toward nonviolent conflict resolution. Specifically, students indicated how they felt about engaging in different behaviors (eg, “Hitting other people,” “Getting into fights with other people”), from *1 = This is completely wrong* to *4 = This is completely okay*. We recoded all items such that higher scores indicated more support for nonviolent conflict resolution strategies. In alignment with the conceptual models, we only used this measure to evaluate the antiextremism lessons.

### Analytic Plan

Originally, we had planned to compare students who received the treatment lessons with those who completed the enhancement lessons; however, after the pilot trial was complete, we noted that both the treatment and enhancement lessons targeted similar mediating variables (eg, resistance skills and decision-making skills) that are at the core of D.A.R.E.’s curricular approach. Given that our pilot analyses used the mediating variables as outcomes, we could not compare the effects between those who received the treatment lessons and those who received the enhancement lessons (ie, students in both conditions received content targeting the key mediating variables). Therefore, for these pilot test analyses, our analytic sample only includes students who received the treatment lessons and we focused on whether there were changes *within* students (pretest vs posttest) rather than *between* students in the treatment and comparison conditions.

Before beginning the main analyses, we first conducted a principal component analysis with varimax rotation for each measure, separately for seventh or eighth grade students (who received the peaceful coexistence lessons) and for ninth grade students (who received the antiextremism lessons). After removing items that did not fit within a unidimensional construct, we computed alphas separately for seventh or eighth grade and ninth grade students at pretest and posttest.

To test whether the treatment lessons changed the targeted mediators and distal outcomes, we estimated a series of 3-level models, to account for the nesting of students (level 1) within classroom (level 2) within schools (level 3); there was 1 D.A.R.E. officer per school, so schools and officer were perfectly confounded. We estimated separate models for each mediator and distal outcome and separate models for seventh eighth grade students and ninth grade students.

## Results

### Pilot Sample Characteristics

Table 1 provides the demographic characteristics of the 1349 students who had both pretest and posttest data and who received the treatment lessons (ie, the analytic sample). About two-third of the sample were boys (note: all schools were single sex). Most of the sample were Bahraini (>96% per grade level) and most of the sample (>94% per grade level) lived with both parents.

**Table 1:** Demographics for analytic sample.

			Peaceful coexistence lessons				Antiextremism lessons
			Grade 7 (n=598), n (%)		Grade 8 (n=256), n (%)		Grade 9 (n=495), n (%)
**Sex**							
	Boy	370 (61.9)		197 (77.0)		298 (60.2)	
	Girl	228 (38.1)		59 (23.0)		197 (39.8)	
**Year of birth**							
	2006 and earlier	5 (0.8)		2 (0.8)		5 (1.0)	
	2007	4 (0.7)		2 (0.8)		447 (90.3)	
	2008	15 (2.5)		238 (93.0)		39 (7.9)	
	2009	533 (89.1)		14 (5.4)		15 (0.0)	
	2010	38 (6.4)		0 (0.0)		1 (0.2)	
	Missing	3 (0.5)		0 (0.0)		3 (0.6)	
**Nationality**							
	Bahraini	580 (97.0)		246 (96.1)		474 (95.8)	
	GCC^a^	1 (0.2)		4 (1.6)		2 (0.4)	
	Arabic	13(2.2)		5 (1.9)		16 (3.2)	
	Other or missing	4 (0.6)		1 (0.4)		3 (0.6)	
**Who do you live with most of the time?**							
	Two parents	564 (94.3)		91.8 (235)		471 (95.2)	
	Mother only	18 (3.0)		16 (6.3)		17 (3.4)	
	Father only	5 (0.8)		3 (1.1)		6 (1.2)	
	Other or missing	11 (1.9)		2 (0.8)		1 (0.2)	

^a^GCC: Gulf Cooperation Council.

### Psychometric Analysis

Each of the targeted mediating variables was a unidimensional construct (ie, all items loaded on a single component). The results from our principal component analyses for the proximal outcomes were less definitive. For empathy, we found that 3-4 components had eigenvalues above 1 (depending on time point or grade) and the items for emotional reactivity loaded across multiple components. When we dropped these 4 items, the remaining items loaded as expected on either cognitive empathy (5 items) or social skills (5 items). Therefore, we only included these 2 subscales in our final analyses. For tolerance, we found that 2 components had eigenvalues above 1, with multiple items cross loading on both components. After examining the component loadings and interitem correlations, we kept 4 items (see Multimedia Appendix 1 for details). For open mindedness toward political and religious views, we found that 2 components had eigenvalues above 1: one component had 3 items that captured open mindedness whereas the other component had 3 items that captured apathy toward political or religious viewpoints; we dropped the latter 3 items from the final scale. For open mindedness toward diversity, we found that 2 components had eigenvalues above 1. The 2 negatively worded items (eg, Religion brings more conflict than peace) loaded on the second component; we dropped these 2 items from the final scale. Finally, the items for attitudes toward nonviolent conflict resolution all loaded on a single construct.

Table 2 provides the Cronbach’s alpha for each of the targeted mediating variables and the proximal outcomes, separately for younger (seventh or eighth grade) and older (ninth grade) students and separately for the pretest and posttest surveys. In general, we found that reliability was higher among older students and higher at posttest.

**Table 2:** Reliability for each scale.

Variable	Items, n	Cronbach α			
		Seventh (pretest)	Ninth (pretest)	Seventh (posttest)	Ninth (posttest)
Descriptive norms about intolerance	7	0.772	0.776	0.821	0.832
Injunctive norms about intolerance	7	0.714	0.774	0.777	0.851
Beliefs about consequences	6	0.855	0.910	0.917	0.932
Resistance skills (self)	4	0.644	0.712	0.745	0.786
Resistance skills (friends)	4	0.688	0.755	0.755	0.822
Decision-making skills (self)	3	0.516	0.553	0.519	0.520
Decision-making skills (friends)	3	0.497	0.583	0.500	0.568
Social skills empathy	5	0.587	0.611	0.671	0.674
Cognitive empathy	5	0.729	0.695	0.789	0.810
Tolerance	4	0.792	0.803	0.855	0.877
Open mindedness toward political and religious views	3	0.637	0.600	0.685	0.657
Open mindedness toward diversity	4	0.713	0.739	0.781	0.801
Attitudes toward nonviolent conflict resolution	6	—^a^	0.867	—	0.870

^a^Not applicable.

### Testing Differences From Pretest to Posttest

Table 3 provides the results for students who received the peaceful coexistence lessons. Compared to pretest, there was a significant decrease in injunctive norms about intolerance, as well as significant increases in beliefs about the consequences of not listening to others, resistance skills self-efficacy (for self and friends), decision-making skills for self, social skills empathy, tolerance beliefs, and open mindedness toward diversity.

**Table 3:** Testing pretest versus posttest differences for seventh and eighth grade students.

Variable	Pretest, mean (95% CI)	Posttest, mean (95% CI)	*P* value	Total, n
Descriptive norms about intolerance	1.91 (1.83-1.99)	1.92 (1.84-2.00)	.80	854
Injunctive norms about intolerance	1.39 (1.34-1.44)	1.31 (1.26-1.35)	<.001	853
Beliefs about consequences	1.87 (1.75-1.98)	1.98 (1.86-2.10)	<.001	850
Resistance skills (self)	2.76 (2.68-2.83)	2.97 (2.89-3.05)	<.001	850
Resistance skills (friends)	2.82 (2.74-2.91)	2.97 (2.88-3.05)	<.001	846
Decision-making skills (self)	3.32 (3.25-3.38)	3.38 (3.31-3.45)	.005	841
Decision-making skills (friends)	3.28 (3.20-3.36)	3.32 (3.24-3.40)	.09	840
Social skills empathy	2.66 (2.61-2.71)	2.73 (2.68-2.78)	.008	830
Cognitive empathy	2.69 (2.62-2.76)	2.74 (2.67-2.81)	.10	827
Tolerance	3.45 (3.36-3.55)	3.50 (3.41-3.60)	.04	828
Open mindedness toward political and religious views	2.87 (2.79-2.96)	2.87 (2.79-2.96)	.95	815
Open mindedness toward diversity	3.27 (3.16-3.38)	3.32 (3.21-3.43)	.02	851

Table 4 provides the results for students who received the anti-extremism lessons. Compared to pretest, there was a significant decrease in injunctive norms about intolerance. There was also a significant increase in beliefs about the consequences of not listening to others, resistance skills self-efficacy for self (but not friends), and social skills empathy.

**Table 4:** Testing pretest versus posttest differences for ninth grade students.

Variable	Pretest, mean (95% CI)	Posttest, mean (95% CI)	*P* value	Total, n
Descriptive norms about intolerance	2.01 (1.93-2.09)	2.04 (1.96-2.12)	.26	495
Injunctive norms about intolerance	1.36 (1.30-1.42)	1.31 (1.25-1.37)	.01	495
Beliefs about consequences	2.20 (2.07-2.34)	2.38 (2.24-2.52)	<.001	494
Resistance skills (self)	2.99 (2.91-3.07)	3.23 (3.15-3.32)	<.001	494
Resistance skills (friends)	3.08 (3.00-3.16)	3.07 (2.99-3.15)	.87	495
Decision-making skills (self)	3.32 (3.24-3.40)	3.37 (3.29-3.45)	.09	495
Decision-making skills (friends)	3.36 (3.28-3.43)	3.38 (3.30-3.46)	.46	495
Social skills empathy	2.61 (2.54-2.67)	2.72 (2.65-2.78)	<.001	490
Cognitive empathy	2.81 (2.72-2.89)	2.84 (2.75-2.93)	.23	490
Tolerance	3.54 (3.45-3.63)	3.50 (3.41-3.59)	.15	490
Open mindedness toward political and religious views	2.89 (2.82-2.97)	2.90 (2.82-2.98)	.86	486
Open mindedness toward diversity	3.36 (3.24-3.48)	3.32 (3.20-3.44)	.12	495
Attitudes toward nonviolent conflict resolution	1.31 (1.26-1.36)	1.32 (1.27-1.37)	.68	485

## Discussion

### Principle Findings

In this manuscript, we reported the results of the preparation phase of the MOST to optimize and evaluate the peaceful coexistence and antiextremism lessons implemented as part of D.A.R.E. (ie, Ma’an) in Bahrain to counter the potential for political and religious extremism and radicalization, which are common in the MENA region. These lessons, intended to be implemented for Middle and High School students in Bahrain, are an important part of the multilevel community approach to reducing extremism, conflict, and associated violence. Countering extremism is an important public health issue, as it helps to increase physical and psychological safety for communities [2-4]. Intervening with adolescents not only attempts to protect a particularly vulnerable population but also aims to prevent radicalization and growth of extremist groups [6,7].

We started by drawing on the existing lessons that Bahrain D.A.R.E. officers had created, prior literature, feedback from US D.A.R.E experts, and local insights from Bahrain D.A.R.E. officers, to develop 2 conceptual models. These models guided our measurement efforts in the current pilot test and will facilitate intervention optimization and program revisions going forward. We then pilot tested our research protocol by evaluating the existing intervention “packages.” The results of this pilot test were encouraging.

First, most of our measures showed evidence of a unidimensional construct and good internal reliability, even though our team had to develop or adapt most of the measures for this study as well as translate them into Arabic. We then used our results to revise the measures further before moving into the optimization phase of MOST. For example, we decided to add and adapt items for measures with lower reliability and to revise or remove most negatively worded items, as these seemed to be particularly problematic for this population. Second, we found that most of the D.A.R.E. officers were able to follow the randomization protocol (even though we ended up not being able to compare the students in the intervention and attention control classrooms), suggesting that they could successfully implement the more complicated protocol for the factorial design that would be used in the optimization phase of our study. Finally, we found initial evidence—described in more detail below—that the existing intervention lessons could change some of the targeted mediating variables and proximal outcomes, giving us a foundation to build on when using the conceptual model to revise the lessons prior to starting the optimization phase of MOST.

In terms of the belief or attitude focused targeted mediators, we found that both students who completed the peaceful coexistence lessons and those who completed the antiextremism lessons reported significantly lower perceived approval of intolerance (ie, injunctive norms about intolerance) at the immediate posttest. By contrast, we found no changes in students’ beliefs about the frequency of peers engaging in intolerant behaviors, such as spreading hateful ideas about people who are different and insulting another persons’ religion (ie, descriptive norms). Some studies suggest that among adults, injunctive norms may be more meaningful than descriptive norms in situations that require recommending whether peers should engage in risky behaviors [30,31]. Still, these studies emphasize the importance of descriptive norms for individual behavioral choices, underscoring the importance of continuing to explore how the lessons could better target descriptive norms. We also found that students who completed either set of lessons reported a significantly higher likelihood that not listening to others would have negative outcomes (ie, beliefs about consequences) at the immediate posttest. With an emphasis on communication, understanding, and empathy [2,12,14], listening is an integral part of peaceful coexistence and an important hypothesized predictor of the proximal outcomes.

In terms of the skill-based targeted mediators, students who completed either set of lessons reported increased efficacy to implement resistance skills at the immediate posttest. In contrast, only the students who received the peaceful coexistence lessons reported significantly higher confidence that they could also encourage their friends to use resistance stills and significantly higher confidence that they could engage in more thoughtful or less impulsive decision-making. Although it is promising that the peaceful coexistence lessons successfully changed self-efficacy toward personal resistance skills and personal decision-making skills, it highlights that something may be missing from the antiextremism curriculum. Given the importance of resistance and decision-making for conflict resolution, a proximal outcome of the antiextremism curriculum in our conceptual model, we plan to explore whether curriculum revisions are needed to improve the antiextremism lessons. Notably, the reliability of the decision-making measures was quite low, suggesting that we also should add or revise items for these measures going forward to better capture any true change in self-efficacy toward decision-making skills over time.

As shown in our conceptual models, we expected that changing the targeted mediators would lead to changes in 3 proximal outcomes in the peaceful coexistence intervention (empathy, tolerance, and open-mindedness) and 4 proximal outcomes in the antiextremism program (empathy, tolerance, and open-mindedness, along with nonviolent conflict resolution skills). The results of our pilot test indicated mixed results: Students who completed the peaceful coexistence lessons reported significantly higher social skills empathy, tolerance beliefs, and open-mindedness toward diversity, but showed no changes in cognitive empathy or open-mindedness about political and religious views. Students who completed the antiextremism lessons reported significant increases in social skills empathy at the immediate posttest but no changes in any of the other proximal outcomes. There are several potential explanations for our mixed results. First, some targeted mediators did not change in response to intervention participation; therefore, the lessons may need to be revised so that they have stronger changes in the targeted mediators before we can expect to see changes in the proximal outcomes. Second, the antiextremism curriculum was originally developed for high school students, but for practical reasons, we had to pilot test the lessons with ninth graders, who are middle school students in Bahrain. Developmental differences may have impacted how the lessons were received. Still, the more limited effects on the proximal outcomes for the antiextremism intervention suggests that we should examine these lessons more during the optimization phase of MOST, with an eye toward revising these lessons going forward.

The results of the preparation phase of MOST led to several conclusions. First, we determined that we would treat each of the lessons as components when we “break apart” the intervention during the optimization phase: In other words, there would be 4 components in the peaceful coexistence program and 4 components in the antiextremism program. Second, we recommended that D.A.R.E. work with the Bahraini team to reconfigure the content of the lessons so that each lesson targeted a single mediator (see the first column in Figures 1 and 2). For example, in the peaceful coexistence program, the lesson about intolerance and video games could be reconfigured to target resistance skills. In contrast, in the existing intervention, the lessons frequently targeted multiple mediators. Reconfiguring the lessons would make it easier to evaluate the individual lessons (components) during the optimization phase of MOST, by allowing us to test whether students who received a particular lesson (eg, the video games lesson) improved more on the targeted mediating variable (eg, resistance skills) compared to students who did not receive that lesson. Third, based on our conversations with stakeholders, it became clear that the most important factor for developing an optimized intervention was identifying the most effective set of lessons possible; there were no time or money constraints that indicated a need to implement a shorter intervention. Therefore, we recommended that the optimization objective for the upcoming optimization trials should be “all effective lessons.” Finally, given the lack of evidence-based interventions targeting peaceful coexistence or antiextremism among adolescents and the desire to build an intervention with clinically meaningful effects, we recommended that the Bahraini team be open to conducting more than 1 optimization trial going forward. Specifically, we suggested that we evaluate the reconfigured lessons in 1 optimization trial, then use the results from that trial to further revise and strengthen lessons with weaker effects, and then test the revised lessons in another optimization trial before creating a “final” intervention package.

### Limitations

Despite the encouraging results from our pilot test, our study had several limitations. First, we selected multiple enhancement lessons for officers to deliver as an “attention control” group. Unfortunately, we realized after the study began that, although the *content* of the lessons differed, these control lessons targeted many of the same mediators, minimizing our ability to detect differences between the treatment and control groups. Therefore, we only analyzed pretest to posttest differences within the treatment group. As with any pretest versus posttest design, any difference could reflect developmental changes over time or changes in response to the survey, rather than changes in attitudes due to the treatment curriculum. Second, as with any design that analyzes an entire “treatment package,” we cannot evaluate the individual lessons. Our results suggest that the curriculums as a whole have statistically significant effects on many of the targeted mediators, but we cannot determine whether each *lesson* had its intended effect, whether any lessons had negative effects that undermine the effects of other lessons. Third, our study did not include any long-term follow-up, which meant we could only focus on changes in attitudes and beliefs, rather than changes in behaviors. Finally, to our knowledge, none of the measures that we used had been used in Bahrain or other Arabic-speaking countries before. Therefore, as with any study using new measures, it is unclear if the lack of effects on some variables was due to a lack of effect from the curriculum or whether it reflected a measurement issue.

### Conclusions

The eventual goal of our project is to develop and optimize peaceful coexistence and antiextremism programs that can eventually be used across the Kingdom of Bahrain and translated for use in other countries to combat the spread of extremism and intolerance. The aim is for these programs to be implemented alongside existing community and policy interventions, allowing for a multilevel and systems approach which is often noted as an essential strategy to countering extremism [4]. This is an important topic for research and intervention in order to protect the health and safety of communities worldwide. Our current study begins to address this critical need by conducting the preparation phase of MOST. Specifically, in this study we (1) developed a conceptual model identifying factors linked to valuing peaceful coexistence and antiextremism behaviors, (2) identified the 4 lessons in each program as the components that we will evaluate in the optimization phase of MOST, (3) pilot tested the existing lessons, our newly developed measures, and research protocol, and (4) determined that our optimization objective going forward will be “all effective components.” Our next step is to conduct the optimization phase of MOST to evaluate, and revise, the individual lessons, so as to be able to eventually create the first ever optimized peaceful coexistence and antiextremism intervention lessons that can be used in other Arabic-speaking countries as well as other countries that implement D.A.R.E.

## Appendix

Multimedia Appendix 1 List of survey items for each scale.

## Abbreviations

D.A.R.E.: Drug Abuse Resistance Education
MOST: multiphase optimization strategy

## Data Availability

The data sets generated during this study are not publicly available due to the sensitivity of the topic and vulnerable population but are available from the corresponding author on reasonable request.
