# Supplementary material for: Using the Preparation Phase of the Multiphase Optimization Strategy to Design an Antiextremism Program in Bahrain: Formative and Pilot Research
Source: JMIR Form Res. 2024 Jul 17;8:e58322. doi: 10.2196/58322 (PMC11292147; doi:10.2196/58322)
Supplement: Multimedia Appendix 1 [file formative_v8i1e58322_app1.docx]

# Appendix

## List of survey items for each scale – Mediating Variables

### Descriptive Norms about Intolerance

*In your opinion, how many kids your age do the following?*

1 = None or almost none, 2 = some, 3 = half, 4 = most, 5 = All or almost all

1. *Spread hateful ideas about people who are different*
2. *Never listen to people who have different opinions*
3. *Try to convince others that their beliefs are the only correct beliefs*
4. *Insult another person's religion*
5. *Act aggressively toward people who are different*
6. *Refuse to obey their parents*
7. *Discriminate against people who are different*

### Injunctive Norms about Intolerance

In your opinion, how do most kids your age feel about other kids doing the following?

1 = It is completely wrong, 2 = It is sort of wrong, 3 = It is sort of okay, 4 = It is completely okay

1. *Spreading hateful ideas about people who are different*
2. *Never listening to people with different opinions*
3. *Trying to convince others that their beliefs are the only correct beliefs*
4. *Insulting another person’s religion*
5. *Acting aggressively toward people who are different*
6. *Refusing to obey their parents*
7. *Discriminating against people who are different*

### Beliefs about Consequences

*How likely or unlikely is it that NOT listening to different opinions will lead to each of the following?*

1 = Very unlikely, 2 = Somewhat unlikely, 3 = Somewhat likely, 4 = Very likely

1. *Lack of respect for the rights and feelings of others*
2. *A lack of acceptance of dialogue between people from different cultures*
3. *People feeling hostile toward others*
4. *The destruction of society*
5. *People losing their rights*
6. *The loss of unity between individuals*

### Resistance Skills (Self)

*If someone tried to persuade YOU to participate in religious, political, or socially extreme activities that you were uncomfortable with, how confident are you that YOU could...*

1 = Not at all confident, 2 = A little confident, 3 = Mostly confident, 4 = Completely confident

1. *Say no.*
2. *Explain why you do not want to participate.*
3. *Keep yourself away from the activities you did not want to participate in.*
4. *Take yourself out of the situation.*

### Resistance Skills (Friends)

*If you saw one of your friends being persuaded to participate in religious, political, or socially extreme activities they were uncomfortable with, how confident are you that YOU could...*

1 = Not at all confident, 2 = A little confident, 3 = Mostly confident, 4 = Completely confident

1. *Help your friend say no.*
2. *Help your friend explain why they do not want to participate.*
3. *Keep your friend away from the activities they did not want to participate in.*
4. *Get your friend out of the situation.*

### Decision-Making Skills (Self)

*In your opinion, how much do YOU disagree or agree with the following statements? When I do something…*

1 = Strongly disagree, 2 = Disagree, 3 = Agree, 4 = Strongly agree

1. *I think carefully about my choices.*
2. *I compare the good things and bad things that might happen.*
3. *I don't even think about it; I just do it (Reverse coded)*

### Decision-Making Skills (Friends)

*In your opinion, how much do YOU disagree or agree with the following statements? I encourage my friends to…*

1 = Strongly disagree, 2 = Disagree, 3 = Agree, 4 = Strongly agree

1. *think carefully about their choices.*
2. *compare the good things and bad things that might happen.*
3. *not even think about it; just do it (Reverse coded)*

## List of survey items for each scale – Proximal Outcome Variables

### Empathy

*We'd like to know more about how YOU feel toward other people and during certain situations. Please choose how much YOU disagree or agree with the following statements.*

1 = Strongly disagree, 2 = Disagree, 3 = Agree, 4 = Strongly agree

1. *I find it difficult to explain things that I understand easily to someone who doesn't understand it the first time.** (social skills)
2. I really enjoy caring for other people.
3. *I find it hard to know what to do in a social situation.** (social skills)
4. *Friendships and relationships are just too difficult, so I tend not to bother with them.** (social skills)
5. *I often find it difficult to figure out if something is rude or polite.** (social skills)
6. *I am good at figuring out how someone will feel.* (cognitive empathy)
7. *I am good at figuring out when someone in a group is feeling uncomfortable*. (cognitive empathy)
8. If someone is offended by what I say, that is their problem, not mine.
9. Seeing people cry doesn't really upset me.
10. *I tend to find social situations confusing.** (social skills)
11. *I can sense if I am intruding, even if the other person doesn't tell me.* (cognitive empathy)
12. *I can quickly figure out how someone else feels.* (cognitive empathy)
13. *I can easily figure out what another person might want to talk about.* (cognitive empathy)
14. I get emotionally involved with my friends' problems.

### Tolerance

*In your opinion, how much do YOU disagree or agree with the following statements? Kids my age should...*

1 = Strongly disagree, 2 = Disagree, 3 = Agree, 4 = Strongly agree

1. *Have respect for other religions.*
2. *Be tolerant to differences.*
3. *Preserve national identity.*
4. Conform to the norms of society.
5. *Listen to their parents and other leaders.*
6. Accept others' opinions without arguing.
7. Be willing to compromise on their beliefs.
8. Recognize that all religions are the same.
9. Question ideas before accepting them as true.
10. Be sensitive to others people's opinions.

### Open Mindedness toward Political and Religious Views

*In your opinion, how much do YOU agree or disagree with the following statements?*

1 = Strongly disagree, 2 = Disagree, 3 = Agree, 4 = Strongly agree

1. *When it comes to politics or religion, I try to reserve judgement until I have a chance to hear arguments from both sides of an issue.*
2. It is a waste of time to pay attention to certain political or religious ideas.
3. *I am open to considering other political or religious viewpoints.*
4. I often "tune out" political or religious messages I disagree with.
5. *When thinking about a political or religious issue, I consider as many different opinions as possible*
6. I have no patience for political or religious arguments I disagree with.

### Open Mindedness toward Diversity

In your opinion, how much do YOU disagree or agree with the following statements?

1 = Strongly disagree, 2 = Disagree, 3 = Agree, 4 = Strongly agree

1. *We must respect all religions.*
2. *All religious groups in Bahrain should have equal rights.*
3. Religion brings more conflict than peace.
4. Religious people are often intolerant of others.
5. *People who have different religious beliefs make my school an interesting place.*
6. *People who come from different countries make my school an interesting place.*

### Attitudes toward Non-Violent Conflict Resolution

*Choose the answer that best describes how YOU feel about doing each of following things.*

1 = This is completely wrong, 2 = This is sort of wrong, 3 = This is sort of okay, 4 = This is completely okay

1. Hitting other people.*
2. Insulting other people.*
3. Saying mean things when you're mad.*
4. Pushing or shoving other people around if you're mad.*
5. Getting into fights with others.*
6. Taking your anger out on others by using physical force.*
